# Supplementary material for: Microbial Populations Are Shaped by Dispersal and Recombination in a Low Biomass Subseafloor Habitat
Source: mBio. 2022 Aug 1;13(4):e00354-22. doi: 10.1128/mbio.00354-22 (PMC9426424; doi:10.1128/mbio.00354-22)
Supplement: FIG S1 [file mbio.00354-22-s0003.pdf]

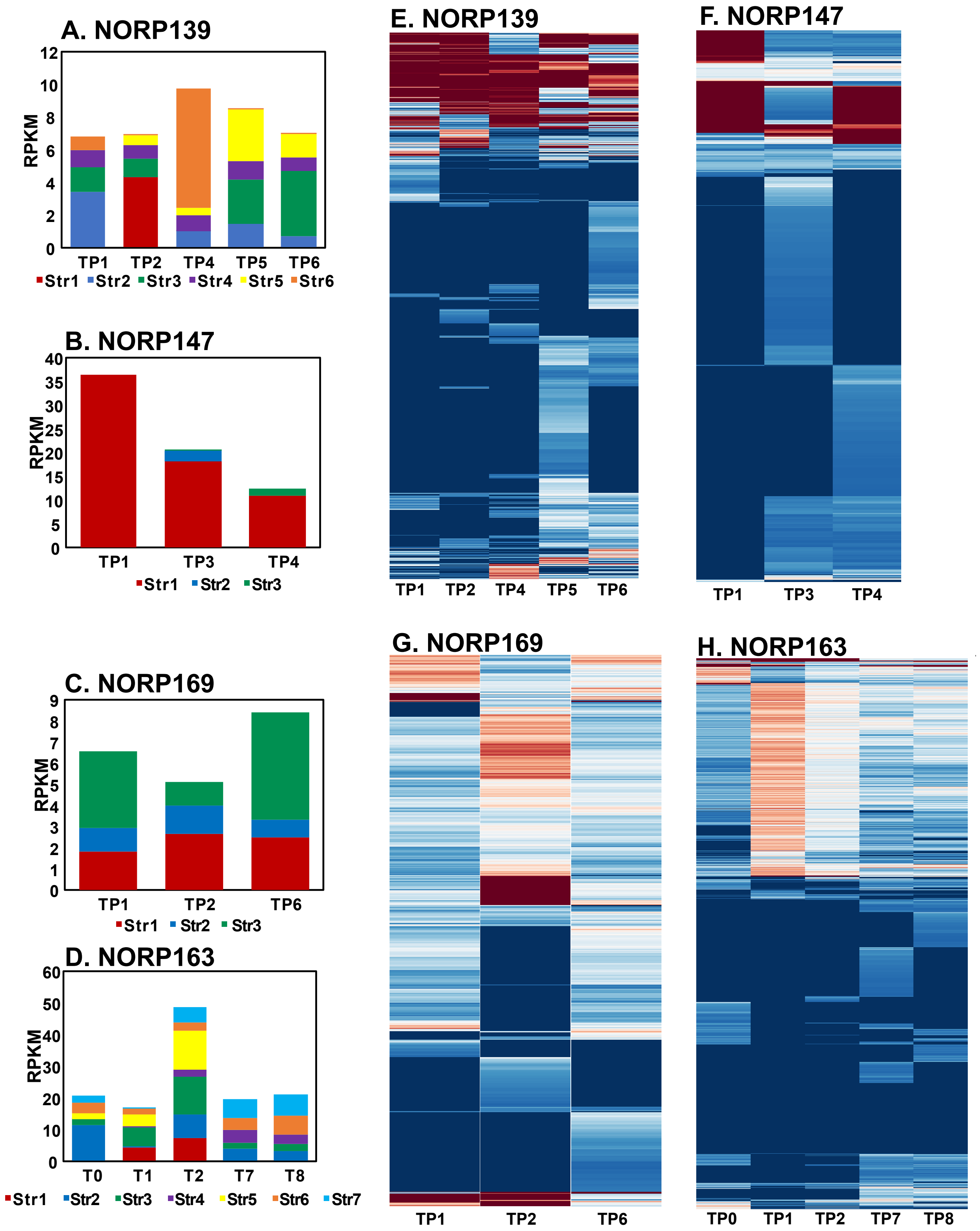

Fig. S1. Strain relative abundance as determined using DESMAN for (A) NORP139, (B) NORP147, (C) NORP169, and (D) NORP163. Major allele frequency for SNVs detected in time points of interest for (E) NORP139, (F) NORP147, (G) NORP169, and (H) NORP163. The major allele frequencies have been hierarchically clustered and scaled from 0-1. Both 0.0 and 1.0 represent a fixed allele; 0.5 represents a split allele.
