# Supplementary material for: Microbial Populations Are Shaped by Dispersal and Recombination in a Low Biomass Subseafloor Habitat
Source: mBio. 2022 Aug 1;13(4):e00354-22. doi: 10.1128/mbio.00354-22 (PMC9426424; doi:10.1128/mbio.00354-22)
Supplement: FIG S2 [file mbio.00354-22-s0002.pdf]

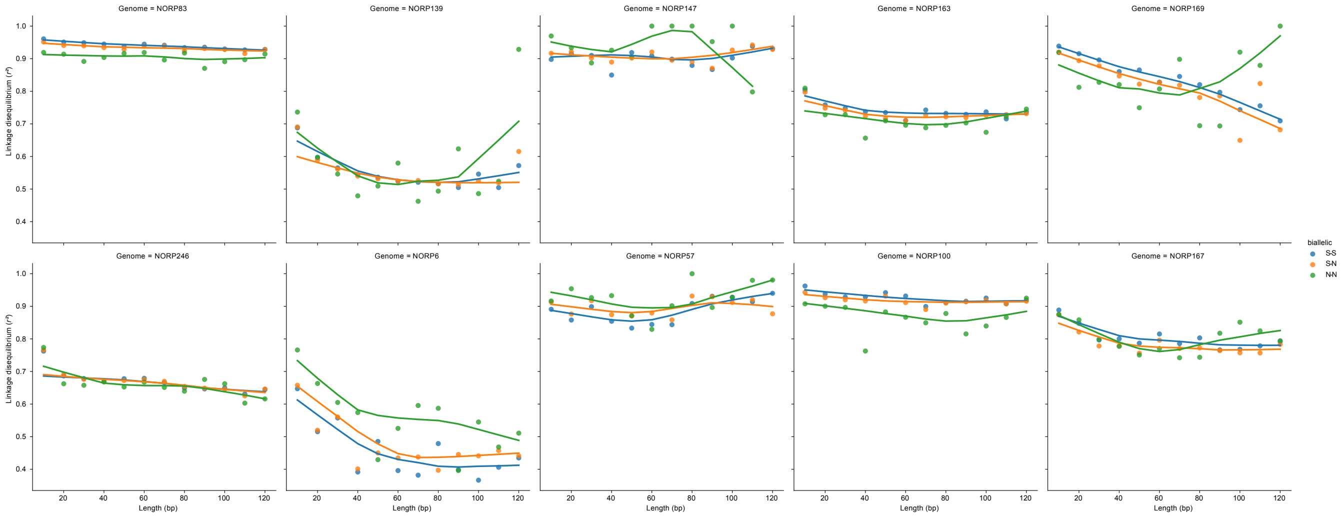

Fig. S2. Linkage disequilibrium of r^2 for linked SNVs pairs for all MAGs. Each circle is the mean r^2 for pairs of linked SNVs at that distance range (*e.g.,* 1-10 bp, 11-20 bp, *etc.*). Linked SNVs are denoted by their predicted mutation type (nonsynonymous: N, synonymous: S).
