# Supplementary material for: Microbial Populations Are Shaped by Dispersal and Recombination in a Low Biomass Subseafloor Habitat
Source: mBio. 2022 Aug 1;13(4):e00354-22. doi: 10.1128/mbio.00354-22 (PMC9426424; doi:10.1128/mbio.00354-22)
Supplement: TABLE S1 [file mbio.00354-22-s0001.pdf]

Table S1. Settings used for each of the 7 passes with Binsanity-lc.

Pass	Preference	Refinement preference	K-means value
1	-25	-50	500
2	-15	-35	50
3	-10	-25	50
4	-5	-25	50
5	-3	-25	50
6	-2	-25	50
7	-2	-10	NA
