# Supplementary material for: Microbial Populations Are Shaped by Dispersal and Recombination in a Low Biomass Subseafloor Habitat
Source: mBio. 2022 Aug 1;13(4):e00354-22. doi: 10.1128/mbio.00354-22 (PMC9426424; doi:10.1128/mbio.00354-22)
Supplement: TABLE S2 [file mbio.00354-22-s0005.pdf]

Table S2. Assembly statistics comparing original 2017 result with modified assembly and binning protocols presented here.

Method	BinSanity approach	No. of contigs $\geq$3kb	No. of contigs $\geq$100kb	N50	No. of MAGs $\geq$50	No. of MAGs $\geq$90
Tully et al. (2018)	pre-release BinSanity	78,004	1,278	25,932	195	68
Tully et al. (2018)	Binsanity-wf	78,004	1,278	25,932	222	67
Method 1	Binsanity-lc	103,964	1,048	19,010	209	96
Method 2	Binsanity-lc	170,001	1,498	17,806	140	46
Method 3	Binsanity-lc	132,025	612	11,653	231	45
